# Post-90-Day Survival After Stroke According to 90-Day Functional Outcome in the 2013 and 2020 Cohorts: A Swedish Stroke Register Study

**DOI:** 10.1159/ned/adwag044

**Published:** 2026-09-30

**Authors:** Marcus Johnsson, Björn M. Hansen, Trine Apostolaki-Hansson, Mats Pihlsgård, Bo Norrving, Teresa Ullberg

**Affiliations:** a Department of Neurology, Skåne University Hospital, Lund, Sweden; b Department of Clinical Sciences Lund, Lund University, Lund, Sweden; c Department of Radiology, Skåne University Hospital, Lund, Sweden; d Stroke Imaging Research Group, Department of Clinical Sciences Lund, Lund University, Lund, Sweden; e Perinatal and Cardiovascular Epidemiology, Department of Clinical Sciences Malmö, Lund University, Lund, Sweden

**Keywords:** Ischemic stroke, ICH, Modified Rankin Scale, 90-day mRS, Long-term survival

## Abstract

**Introduction:**

Ninety-day functional outcome is a strong predictor of long-term survival after stroke, but whether survival differs across calendar-year cohorts within a national stroke population at similar levels of poststroke disability remains unclear. We therefore examined whether long-term survival from 90 days to 3 years after stroke differed between 2013 and 2020, stratified by functional outcome at 90 days.

**Methods:**

In this nationwide register-based cohort study using the Swedish Stroke Register, we included patients with ischemic stroke (IS) or intracerebral hemorrhage (ICH) admitted in 2013 or 2020 who were prestroke independent (modified Rankin Scale [mRS] 0–2) and survived 90 days. The primary outcome was all-cause mortality between day 90 and 3 years after stroke. Multivariable Cox regression models estimated adjusted hazard ratios (aHRs) and assessed whether the association between calendar-year cohort and mortality differed by 90-day mRS.

**Results:**

Among 27,452 patients (24,574 IS and 2,878 ICH), adjusted 3-year survival improved for 90-day survivors in 2020 compared with 2013 after IS (aHR 0.86, 95% CI 0.80–0.92), whereas no improvement was observed after ICH (aHR 0.98, 95% CI 0.80–1.19). In IS, higher survival in 2020 was observed both among functionally independent patients (mRS 0–2; aHR 0.76, 95% CI 0.68–0.86; 3-year survival 93.4% vs. 91.9%) and those with dependency at 90 days (mRS 3–5; aHR 0.84, 95% CI 0.77–0.91; 76.3% vs. 71.7%). In contrast, survival was not significantly different after ICH (mRS 0–2: 90.7% vs. 91.1%; mRS 3–5: 78.5% vs. 77.8%). Across both stroke subtypes, 90-day disability remained strongly associated with long-term mortality.

**Discussion:**

Post-90-day survival after IS was higher in the 2020 than the 2013 cohort and was observed across the full spectrum of poststroke disability. Findings suggest that gains in survival are not limited to patients with favorable recovery but extend across levels of poststroke disability, potentially reflecting improvements in post-acute stroke care and secondary prevention. In contrast, no corresponding improvement was observed across functional outcome levels after ICH. Further studies are needed to confirm these findings and investigate their underlying mechanisms.

## Plain Language Summary

Stroke can lead to long-term disability and may affect survival for several years. In this nationwide Swedish study, we followed more than 27,000 people who were alive 90 days after a stroke and compared patients treated in 2013 with those treated in 2020. After ischem.ic stroke (IS), caused by a blocked blood vessel in the brain, survival during the following 3 years was better in 2020 than in 2013. Importantly, this improvement was seen both in patients who had recovered well and in those who still had disability 90 days after their stroke. Previous studies have shown that disability after stroke is linked to long-term survival, but it has not been clear whether survival has improved over time for patients with different levels of recovery. In contrast, survival after intracerebral hemorrhage, caused by bleeding in the brain, was similar in 2013 and 2020. These findings show that improved long-term survival after IS was not only limited to patients with a good recovery but was also seen in those with remaining disability.

## Introduction

Stroke is a leading cause of death and long-term disability worldwide [1]. Although early mortality remains substantial, survival during the acute phase has improved over recent decades, particularly after ischemic stroke (IS) [2–4]. As a consequence, an increasing proportion of patients survive the acute phase, shifting clinical focus toward long-term outcomes among post-acute survivors.

Ninety-day functional outcome, commonly assessed by the modified Rankin Scale (mRS), is a well-established predictor of long-term mortality after stroke. Earlier studies have demonstrated a graded association between increasing mRS levels and subsequent risk of death [5–9]. However, these studies largely reflect earlier treatment eras, 1980s to early 2000s, and primarily describe the prognostic impact of disability within single cohorts, without addressing how survival patterns may have changed over time within the different mRS strata.

Swedish nationwide data have shown lower long-term mortality in 2020 compared with 2013 after IS but not after intracerebral hemorrhage (ICH) [10]. However, contemporary data on post-acute survival among patients who survive the initial poststroke period remain limited. Furthermore, it remains unclear whether between-cohort differences in survival are observed across all levels of poststroke disability or are confined to patients with favorable functional recovery.

Using nationwide data from the Swedish Stroke Register (Riksstroke), we compared 3-year survival among 90-day stroke survivors between the 2013 and 2020 cohorts across levels of functional outcome at 90 days to determine whether differences in survival over time varied according to poststroke disability and stroke subtype.

## Methods

### Study Design and Data Sources

This nationwide register-based cohort study evaluated whether long-term survival differed between the 2013 and 2020 cohorts across levels of functional status at 90 days, and between stroke subtypes (IS and ICH). Data were derived from Riksstroke, the Swedish national quality register for acute stroke care, which prospectively collects standardized clinical information from all hospitals providing acute stroke services in Sweden. Register coverage was high, capturing approximately 91% and 87% of hospitalized stroke cases nationwide in 2013 and 2020, respectively. The study population was drawn from the national Riksstroke cohorts for 2013 and 2020 and included patients admitted during the respective calendar years [11, 12]. Since Riksstroke was established in 1994, only two national long-term follow-ups with 3-year outcome ascertainment have been conducted for patients registered in 2013 and 2020. Routine Riksstroke follow-up otherwise extends to 1 year after stroke. Mortality data, however, were available independently through national register linkage and were not the factor determining selection of these two calendar-year cohorts. The study should therefore be interpreted as a comparison between two discrete calendar-year cohorts rather than as an analysis of temporal trends over the intervening years. Sweden’s population was 9.64 million at the end of 2013 and 10.38 million at the end of 2020. Registered immigration and emigration were 115,845 and 50,715 in 2013, compared with 82,518 and 48,937 in 2020 (Statistics Sweden) [13]. Vital status and exact dates of death were obtained through continuous linkage with the Swedish Cause of Death Register, providing near-complete ascertainment of mortality through national register linkage [14].

### Participants and Follow-Up

Eligible participants were adults (≥18 years) registered in Riksstroke with a primary diagnosis of IS (ICD-10 I63) or spontaneous ICH (ICD-10 I61) during calendar years 2013 or 2020. For individuals with more than one registered stroke admission within the same calendar year, only the first event was included. To define a clinically coherent post-acute survivor cohort, analyses were restricted to patients who were functionally independent prior to stroke, defined as prestroke mRS 0–2, and who survived at least 90 days after the index stroke. Patients with prestroke dependence (mRS 3–5) or insufficient information to determine prestroke functional status were excluded. This restriction improves interpretability of poststroke functional outcome strata but may limit generalizability to patients with prestroke dependency.

Information on functional outcome at 90 days was obtained from the standardized Riksstroke 3-month follow-up survey, distributed by mail with two postal reminders to nonrespondents, by telephone interview, or during an outpatient visit, and completed by the patient alone, with caregiver assistance, or by a caregiver alone. Ninety-day mRS was reconstructed using a validated translation algorithm based on predefined decision rules derived from five activities of daily living items: living conditions, dressing, toileting, mobility, and dependency on next of kin for support. In the original validation study of 555 patients, the five-item translation achieved 76% agreement with the reference mRS classification (correlation 0.821; weighted kappa 0.853) [15]. Because the algorithm does not permit separation of mRS grades 0, 1, and 2, mRS 0–2 was analyzed as a single category representing functional independence, whereas mRS 3, 4, and 5 were analyzed separately. Individuals who did not return the questionnaire or lacked sufficient information for mRS reconstruction were classified as having missing 90-day mRS. The same functional items and decision rules were applied to reconstruct prestroke mRS (0–5) from baseline Riksstroke data. Follow-up for mortality began on day 90 after the index stroke and continued until death or censoring at 3 years. The 3-year follow-up horizon was chosen to capture survival beyond the early recovery period while maintaining comparable follow-up duration for both cohorts. For patients with partially missing index dates, where month and year were known but the day was missing, the date was imputed as the 15th day of the respective month.

### Exposure, Outcome, Functional Stratification, and Covariates

Covariates for model adjustment were prespecified and extracted from standardized Riksstroke registry items. Demographic characteristics included age, analyzed as a continuous variable, sex, and living situation. Vascular risk factors at admission included previous stroke, previous transient ischemic attack or amaurosis fugax, atrial fibrillation (AF), hypertension, diabetes mellitus, and current smoking status. A separate diagnosis of hyperlipidemia was not available in the analytical dataset. Statin use at stroke onset was reported descriptively but was not treated as equivalent to a diagnosis of hyperlipidemia. Previous stroke was recorded using the standardized acute Riksstroke item (yes/no, with missing information recorded separately). The analytical extract did not specify whether previous stroke history had been established locally from patient or proxy report, medical-record review, imaging, or another source.

Medication use at admission and discharge included antiplatelet therapy and oral anticoagulant (OAC) therapy. Stroke severity at admission was assessed using the National Institutes of Health Stroke Scale (NIHSS). Level of consciousness at admission was also recorded according to the Reaction Level Scale (RLS-85), categorizing patients as alert (RLS 1), drowsy (RLS 2–3), or comatose (RLS 4–8); the RLS-85 is widely used in Swedish clinical practice and correlates strongly with the Glasgow Coma Scale, which improves comparability with studies reporting consciousness level using the Glasgow Coma Scale [16]. Hospital care variables included stroke unit admission and length of hospital stay. In patients with IS, acute treatments included intravenous thrombolysis, endovascular thrombectomy, and decompressive hemicraniectomy.

### Statistical Analysis

All analyses were conducted using IBM SPSS Statistics version 30.0 (IBM Corp.) and R version 4.5.2. Categorical variables were presented as frequencies with percentages and continuous variables as medians with interquartile ranges. Survival time was calculated from day 90 after the index stroke and continued until death or censoring at 3 years. Kaplan-Meier survival curves were generated for each 90-day mRS category, and differences in survival curves between calendar years were assessed using stratum-specific log-rank tests. Analyses were performed separately for IS and ICH.

Multivariable Cox proportional hazards regression models were used to estimate adjusted hazard ratios (aHRs) with 95% CIs for all-cause mortality from day 90 to 3 years after stroke. The primary exposure variable was calendar-year cohort (2020 vs. 2013). Functional outcome at 90 days was prespecified as the main functional stratification variable and a potential effect modifier, categorized as mRS 0–2 (reference), 3, 4, and 5. Covariates were selected a priori based on clinical relevance and previous stroke outcome studies and were introduced sequentially in predefined hierarchical models to evaluate the stability of the calendar-year effect under progressive adjustment. Model 1 included calendar year and 90-day mRS. Model 2 additionally adjusted for age and sex. Model 3 further included selected vascular risk factors (diabetes mellitus, AF, and hypertension) [17]. The primary estimate of interest was the adjusted association between calendar-year cohort and mortality from day 90 to 3 years. Coefficients for adjustment covariates are presented to document model specification and should not be interpreted as independent causal effects. Within the study design, functional outcome at 90 days was considered to reflect the combined effects of initial stroke severity, acute treatment, and early recovery. These factors were therefore not included separately in the Cox regression models, as their influence was assumed to be captured by the 90-day mRS.

To evaluate whether the association between calendar year and mortality differed across levels of functional outcome, interaction terms between calendar year and 90-day mRS were included in the fully adjusted Cox models. Primary analyses used a dichotomized functional outcome (mRS 0–2 vs. 3–5). Additional analyses were performed using the full mRS classification (0–2, 3, 4, and 5) to allow a more detailed assessment of potential differences across disability levels. Calendar-year-specific hazard ratios (2020 vs. 2013) were derived for each functional stratum from these models. Covariate estimates other than calendar-year cohort and 90-day mRS are presented to document adjustment and should not be interpreted as independent causal effects. The proportional hazards assumption was evaluated using scaled Schoenfeld residuals. In the present analysis, some covariates showed evidence of nonproportionality; however, Cox proportional hazards models were retained, and hazard ratios were interpreted as average effects over the follow-up period. All statistical tests were two-sided and a *p* value <0.05 was considered statistically significant.

#### Missing Data

Missing data in 90-day mRS were handled using multiple imputation by chained equations under a missing-at-random assumption conditional on observed covariates and outcome information [18, 19]. The imputation model included discharge destination, calendar year, survival time, age, sex, admission stroke severity (level of consciousness), vascular risk factors (smoking, diabetes mellitus, hypertension, AF, and previous stroke), and secondary prevention variables (OACs, antiplatelet therapy, and statins). In the IS cohort, intravenous thrombolysis and endovascular thrombectomy were additionally included because of their prognostic relevance. Earlier studies have shown that discharge destination is a particularly informative predictor of poststroke disability at 90 days [20]. Missingness for this variable was low in our dataset (<2.3%). Plausibility of the missing-at-random assumption was assessed descriptively by comparing patients with and without 90-day follow-up (online suppl. Table S2; for all online suppl. material, see https://doi.org/10.1159/ned/adwag044).

Imputations were performed separately for IS and ICH to account for clinical heterogeneity between stroke subtypes, and calendar year was included as a predictor in the imputation models. Twenty imputed datasets were generated, and Cox regression models were estimated within each dataset with parameter estimates combined using Rubin’s rules according to established methodological recommendations [18]. Complete-case analyses were conducted as sensitivity analyses. Additional complete-case sensitivity analyses added previous stroke and then current smoking to Model 3. Smoking was evaluated in these analyses because its missingness differed between cohorts (IS: 5.2% in 2013 and 11.4% in 2020; ICH: 7.5% and 15.8%, respectively). Exploratory complete-case analyses in IS estimated the calendar-year association with mortality separately by AF status, with a calendar-year-by-AF interaction and additional NIHSS adjustment. Among patients with IS and AF, admission NIHSS was summarized by OAC use at stroke onset and calendar year.

### Ethics and Reporting

The study was approved by the Swedish Ethical Review Authority (Dnr 2024-08008-01) and conducted in accordance with the Declaration of Helsinki [21]. In accordance with Swedish regulations for national quality registers, individual informed consent was not required; however, patients are informed about registration and that data may be used for research purposes and have the opportunity to opt out. Reporting followed the Strengthening the Reporting of Observational Studies in Epidemiology (STROBE) guidelines [22]. OpenAI ChatGPT (GPT-5.5; last accessed July 5, 2026) was used solely for language editing and refinement after the authors had independently prepared the initial draft of the manuscript.

## Results

### Study Population and Baseline Characteristics

The final 90-day cohort comprised 14,601 patients in 2013 (1,566 with ICH and 13,035 with IS) and 12,851 patients in 2020 (1,312 with ICH and 11,539 with IS). Overall, 27,452 patients were included across both calendar years (2,878 ICH and 24,574 IS). The study inclusion flow-chart is shown in online supplementary Figure S1.

Baseline characteristics are shown in Table 1. Among patients with IS, mean (SD) age was 71.9 (12.0) years in 2013 and 72.0 (12.0) years in 2020; women comprised 5,506 (42.2%) and 4,681 (40.6%) of the respective cohorts. Among patients with ICH, mean (SD) age was 68.4 (13.4) and 68.3 (14.3) years, and women comprised 654 (41.8%) and 527 (40.2%), respectively. Vascular risk factor profiles were largely comparable between the cohorts. OAC use at admission increased between the study periods. Stroke unit admission and the use of endovascular thrombectomy increased and median hospital length of stay decreased over time.

**Table 1.** Baseline Characteristics of Prestroke-Independent 90-Day Stroke Survivors in the 2013 and 2020 Cohorts

​	Ischemic stroke			Intracerebral hemorrhage		​
variable	2013 (*n* = 13,035)	2020 (*n* = 11,539)	*p* value	2013 (*n* = 1,566)	2020 (*n* = 1,312)	*p* value
Female (%)	5,506 (42.2)	4,681 (40.6)	0.008	654 (41.8)	527 (40.2)	0.386
Age, years (SD)	71.9 (12.0)	72.0 (12.0)	0.750	68.4 (13.4)	68.3 (14.3)	0.948
Living alone (%)	4,978 (38.2)	4,207 (36.5)	0.008	541 (34.5)	429 (32.7)	0.364
Risk factors						
Prior stroke (%)	2,082 (16.0)	1,621 (14.0)	<0.001	229 (14.6)	174 (13.3)	0.270
Previous TIA/amaurosis fugax (%)	1,025 (7.9)	973 (8.4)	0.149	94 (6.0)	74 (5.6)	0.653
Atrial fibrillation (%)	2,981 (22.9)	2,652 (23.0)	0.989	277 (17.7)	248 (18.9)	0.446
Hypertension (%)	7,770 (59.6)	7,022 (60.9)	0.105	824 (52.6)	688 (52.4)	0.743
Smoking (%)^b^	2,104 (16.1)	1,616 (14.0)	0.013	196 (12.5)	143 (10.9)	0.661
Diabetes mellitus (%)	2,657 (20.4)	2,551 (22.1)	0.001	246 (15.7)	192 (14.6)	0.400
Prior statin use (%)	3,768 (28.9)	3,822 (33.1)	<0.001	361 (23.1)	351 (26.8)	0.026
Antiplatelet therapy at admission						
Single antiplatelet therapy (%)	4,000 (30.7)	2,957 (25.6)	<0.001	292 (18.6)	216 (16.5)	0.076
Dual antiplatelet therapy (%)	144 (1.1)	158 (1.4)	0.068	16 (1.0)	4 (0.3)	0.021
OAC therapy at admission (%)	1,138 (8.7)	1,467 (12.7)	<0.001	226 (14.3)	250 (19.1)	<0.001
Direct-acting OAC (%)	99 (0.8)	1,072 (9.3)	<0.001	16 (1.0)	174 (13.3)	<0.001
Vitamin K antagonist (%)	1,039 (8.0)	395 (3.4)	<0.001	210 (13.4)	76 (5.8)	<0.001
Clinical information						
NIHSS^a^ at admission median (IQR)	2 (1–6)	2 (1–5)	<0.001	4 (1–9)	4 (1–11)	0.104
Level of consciousness at admission	​	​	0.298	​	​	0.157
Alert (%)	12,096 (92.8)	10,800 (93.6)	​	1,239 (79.1)	1,011 (77.1)	​
Drowsy (%)	659 (5.1)	554 (4.8)	​	236 (15.1)	234 (17.8)	​
Comatose (%)	118 (0.9)	89 (0.8)	​	64 (4.1)	55 (4.2)	​
Stroke unit admission (%)	9,544 (73.2)	8,897 (77.1)	<0.001	940 (60.0)	792 (60.4)	0.996
Length of hospital stay, days (IQR)	8 (4–17)	6 (3–12)	<0.001	19 (8–39)	15 (7–34)	<0.001
Acute treatments						
Intravenous thrombolysis (%)	1,618 (12.4)	1,685 (14.6)	<0.001	–	–	–
Endovascular thrombectomy (%)	177 (1.4)	637 (5.5)	<0.001	–	–	–
Hemicraniectomy due to IS (%)	40 (0.3)	36 (0.3)	0.928	–	–	–

Data presented as numbers (%), mean (SD), or median (IQR).

IS, ischemic stroke; ICH, intracerebral hemorrhage; mRS, modified Rankin Scale; NIHSS, National Institutes of Health Stroke Scale; OAC, oral anticoagulant; TIA, transient ischemic attack.

^a^ NIHSS had substantial missingness (IS: 44.3% in 2013 and 27.4% in 2020; ICH: 57.8% and 37.8%, respectively).

^b^ Missingness was generally below 1% for baseline variables, except for current smoking, which had 5.2% and 11.4% missing data in IS in 2013 and 2020, respectively, and 7.5% and 15.8% in ICH. Missingness was <1.7% for level of consciousness and <3% for length of hospital stay.

Secondary preventive treatments at discharge are shown in Figure 1 and online supplementary Table S1. Among patients with IS and AF, OAC use increased substantially, particularly among those aged ≥80 years, rising from 62.0% (834/1,345) in 2013 to 87.1% (1,015/1,165) in 2020. Similarly, statin use increased across all subgroups, with the largest absolute increases observed among older patients, increasing from 64.0% (2429/3,794) in 2013 to 82.5% (2664/3,228) in 2020 among those aged ≥80 years. In contrast, antihypertensive treatment rates remained relatively stable over time in both IS and ICH.

**Fig. 1.**
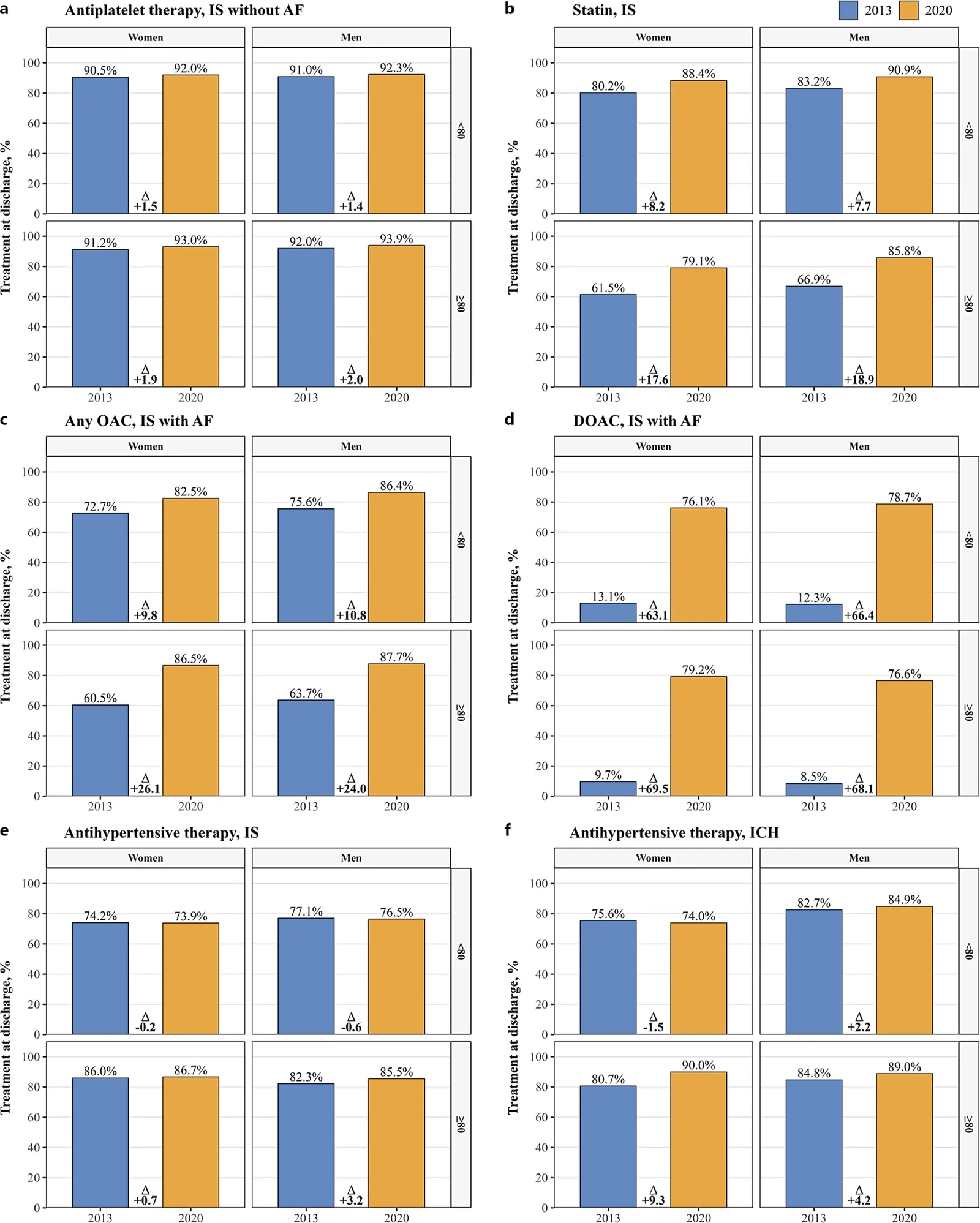
Differences in discharge secondary preventive treatment patterns between the 2013 and 2020 cohorts, stratified by sex and age. The panels show proportions of patients receiving treatment in 2013 and 2020, stratified by sex and age (<80 and ≥80 years). (**a**) Antiplatelet therapy in ischemic stroke (IS) without atrial fibrillation (AF). (**b**) Statin therapy in all IS. (**c**) Any oral anticoagulant (OAC) in IS with AF. (**d**) Direct oral anticoagulant (DOAC) use in IS with AF. (**e**) Antihypertensive treatment in IS. (**f**) Antihypertensive treatment in intracerebral hemorrhage (ICH). Bars represent percentages, and Δ indicates absolute percentage-point differences between 2013 and 2020.

Baseline characteristics among patients with IS, stratified by 90-day functional outcome, are presented in online supplementary Table S6. Within each mRS stratum, patients in 2013 and 2020 were broadly comparable in age, sex, stroke severity, and major vascular risk factors, with age and NIHSS remaining essentially unchanged over time. Modest differences included lower smoking prevalence in mRS 0–2 and higher rates of diabetes mellitus and prior statin use in mRS 3–5 in 2020. In contrast, treatment-related variables changed more substantially, with increased stroke unit admission, greater use of OAC and endovascular thrombectomy, and shorter hospital stays.

Overall, 90-day mRS was missing in 4,410 of 27,452 patients (16.1%) prior to multiple imputation. Online supplementary Figure S2 presents the full distribution of 90-day mRS categories before and after multiple imputation. Missing data for 90-day mRS were 12.6% in 2013 and 18.4% in 2020 among patients with IS, and 17.4% in 2013 and 28.8% in 2020 among patients with ICH. Missingness was higher in ICH and in the 2020 cohort. Patients without 90-day follow-up were younger but had indicators of more severe stroke compared with those who were followed up (online suppl. Table S2). After imputation, the overall distribution of functional outcome categories remained largely comparable between the two calendar-year cohorts.

### 90-day Disability and Long-Term Survival

Higher levels of disability at 90 days were associated with progressively lower long-term survival and higher mortality (Fig. 2a–d). Observed mortality from day 90 to 3 years after the index stroke increased across higher 90-day mRS categories in both stroke subtypes (Table 2). Patients with mRS 0–2 had the most favorable survival, whereas each successive increase in mRS category was accompanied by a stepwise decline in survival. This graded association between disability and long-term survival was consistent across both IS and ICH and showed a similar overall pattern in the 2013 and 2020 cohorts. Comparisons of survival are shown in Figure 3. In IS (Fig. 3a, b), 3-year survival after 90 days was higher in the 2020 cohort compared with 2013, both among patients with favorable functional outcome (mRS 0–2; 93.4% vs. 91.9%) and among those with residual disability (mRS 3–5; 76.3% vs. 71.7%). The largest absolute difference was observed among patients with residual disability (mRS 3–5). Complete-case sensitivity analyses yielded similar Kaplan-Meier curves (online suppl. Fig. S4). In contrast, no difference in survival was observed in ICH (Fig. 3c, d), with 3-year survival remaining stable between 2013 and 2020 among both patients with mRS 0–2 (90.7% vs. 91.1%) and mRS 3–5 (78.5% vs. 77.8%). Kaplan-Meier curves including patients with missing 90-day mRS (online suppl. Fig. S3) showed a modest calendar-year difference in IS, whereas no corresponding difference was evident in ICH. Survival among patients with missing 90-day mRS is presented in online supplementary Figure S5.

**Fig. 2.**
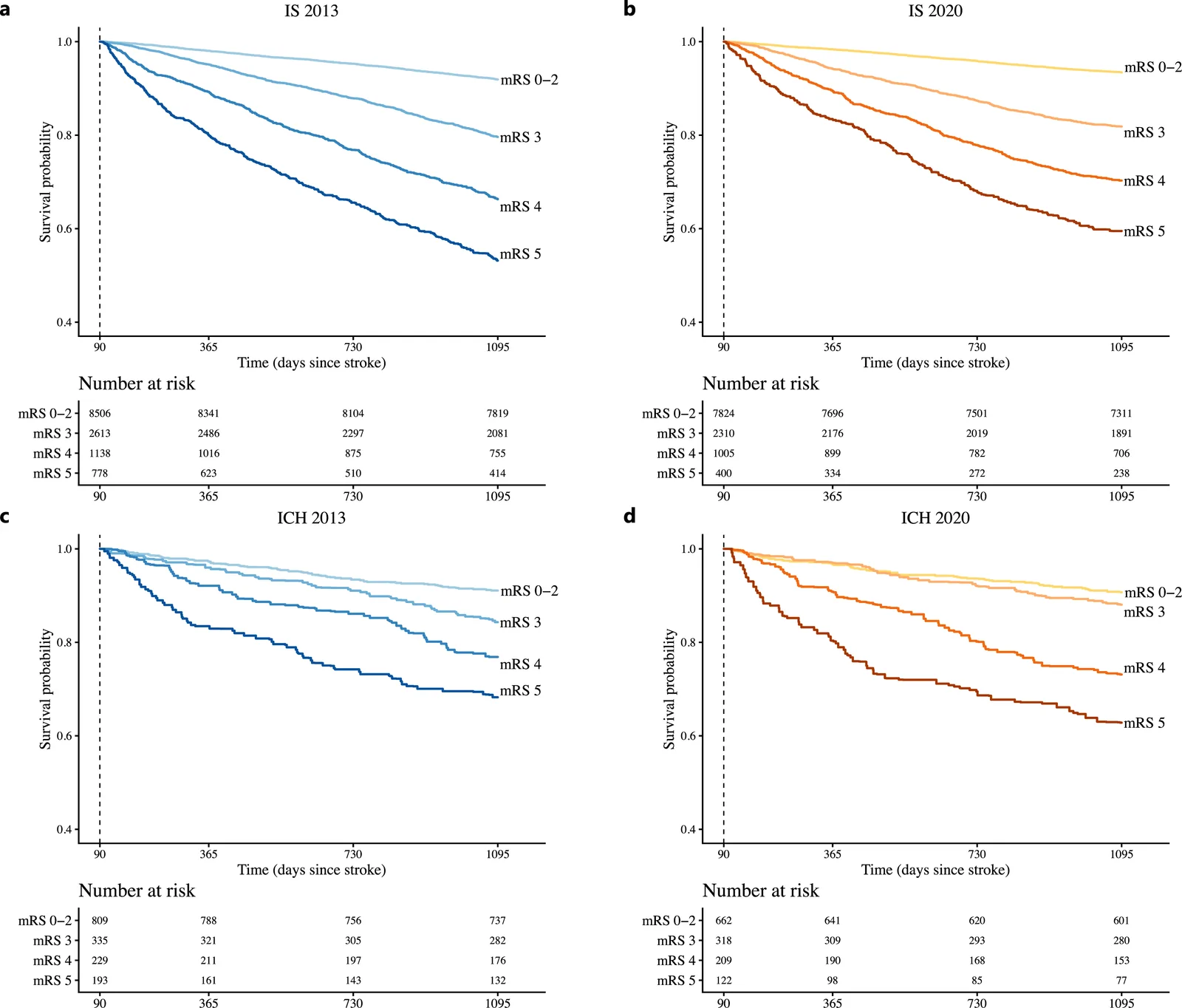
Kaplan-Meier survival from 90 days to 3 years stratified by 90-day mRS. Panels show ischemic stroke in 2013 (**a**) and 2020 (**b**) and intracerebral hemorrhage in 2013 (**c**) and 2020 (**d**). Survival time was calculated from day 90 after stroke and analyses were based on multiply imputed data. This figure is intended to show the graded association between 90-day disability and subsequent survival within each cohort. Between-cohort comparisons within disability strata are shown in Fig. 3. The y-axis is truncated at 0.4 to improve visualization of differences between groups.

**Table 2.** Raw 3-Year Mortality from Day 90 After Stroke According to 90-Day Functional Outcome

90-day functional outcome	IS 2013	IS 2020	ICH 2013	ICH 2020
Overall	1,969/13,035 (15.1%)	1,394/11,539 (12.1%)	240/1,566 (15.3%)	201/1,312 (15.3%)
mRS 0–2	572/7,512 (7.6%)	391/6,471 (6.0%)	59/679 (8.7%)	40/486 (8.2%)
mRS 3–5	1,029/3,887 (26.5%)	654/2,944 (22.2%)	132/615 (21.5%)	84/448 (18.8%)
mRS 3	437/2,285 (19.1%)	322/1,876 (17.2%)	45/282 (16.0%)	23/229 (10.0%)
mRS 4	309/975 (31.7%)	220/785 (28.0%)	41/182 (22.5%)	37/144 (25.7%)
mRS 5	283/627 (45.1%)	112/283 (39.6%)	46/151 (30.5%)	24/75 (32.0%)

Values are deaths/number of patients (%). Overall estimates include all 90-day survivors, whereas mRS-specific estimates are restricted to patients with observed 90-day mRS. The dichotomized mRS 0–2 vs. 3–5 classification represents the primary functional stratification; individual mRS 3, 4, and 5 categories are shown for additional detail.

**Fig. 3.**
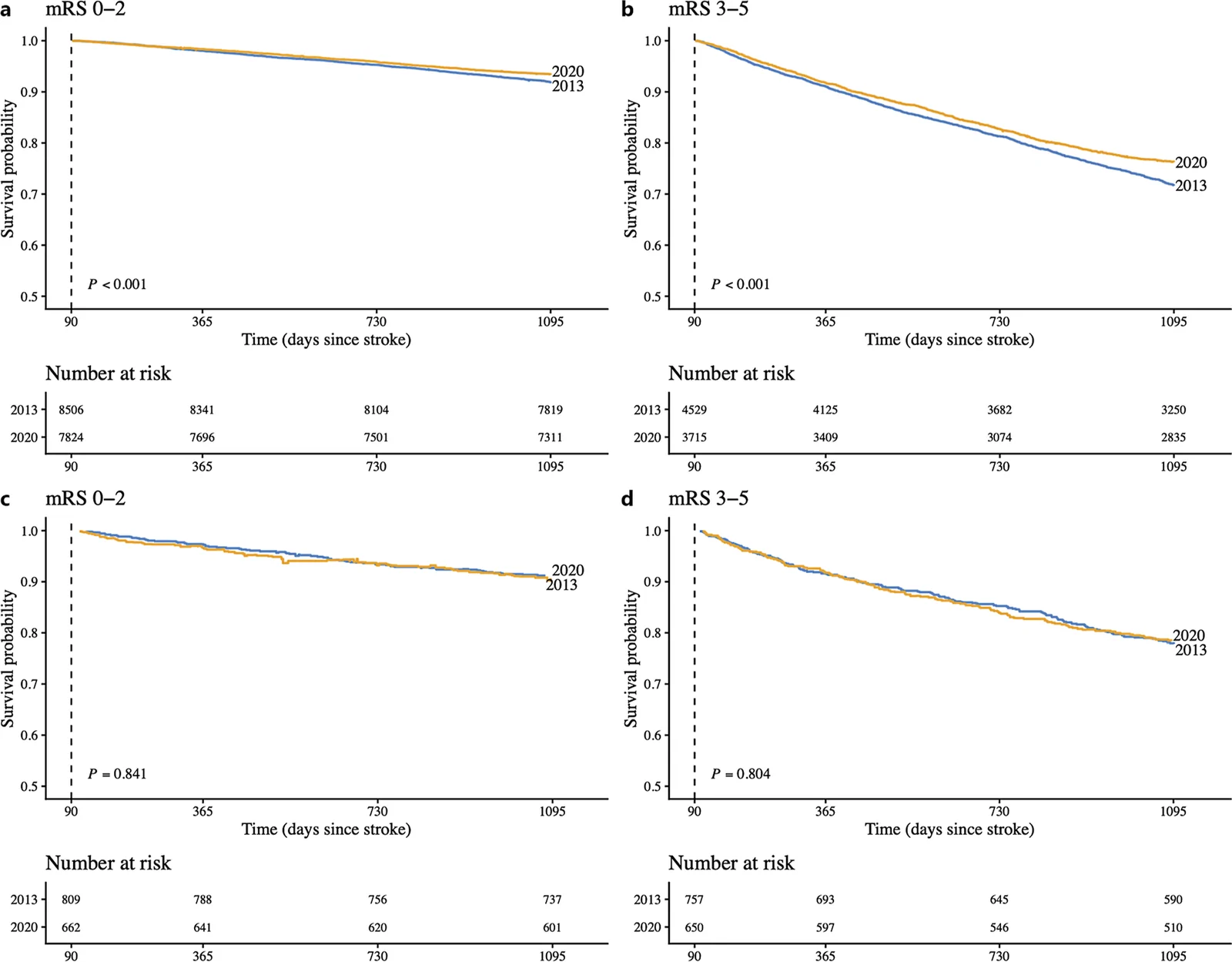
Kaplan-Meier survival from 90 days to 3 years by calendar year within strata of 90-day disability. Panels (**a**) and (**b**) show ischemic stroke and panels (**c**) and (**d**) intracerebral hemorrhage; panels (**a**) and (**c**) show mRS 0–2 and panels (**b**) and (**d**) show mRS 3–5. Curves compare the 2013 and 2020 cohorts and are based on multiply imputed data. Three-year survival after ischemic stroke was 91.9% in 2013 and 93.4% in 2020 among patients with mRS 0–2, and 71.7% in 2013 and 76.3% in 2020 among those with mRS 3–5. Corresponding survival after intracerebral hemorrhage was 91.1% and 90.7% for mRS 0–2, and 77.8% and 78.5% for mRS 3–5, respectively. *p* values are derived from pooled log-rank tests across the 20 imputed datasets. The y-axis is truncated at 0.5 to improve visualization of differences between groups.

### Cox Proportional Hazards Regression

In fully adjusted Cox models using imputed data (Table 3), higher 90-day disability was associated with progressively worse long-term survival in both stroke subtypes. Calendar-year analyses showed higher survival after IS in the 2020 cohort compared with 2013. After full adjustment, patients with IS in 2020 had lower long-term mortality than those in 2013 (aHR 0.86, 95% CI 0.80–0.92; *p* < 0.001), whereas no independent calendar-year effect was observed in ICH (aHR 0.98, 95% CI 0.80–1.19). Stratified analyses showed lower mortality in 2020 across functional outcome strata in IS (Fig. 4). In the primary dichotomized analysis, lower mortality in 2020 was observed both among patients with mRS 0–2 (aHR 0.76, 95% CI 0.68–0.86; *p* < 0.001) and those with mRS 3–5 (aHR 0.84, 95% CI 0.77–0.91; *p* < 0.001). Analyses using the full mRS classification showed lower mortality in 2020 among patients with mRS 0–2, whereas differences between calendar years were not statistically significant within individual higher disability categories (mRS 3, mRS 4, and mRS 5). No calendar-year differences were observed in ICH. In the primary dichotomized model (mRS 0–2 vs. 3–5), no interaction between calendar year and functional status was observed for IS (*p* for interaction = 0.253) or ICH (*p* for interaction = 0.634). Similarly, analyses using the four-category mRS classification showed no statistically significant interaction across disability strata for IS or ICH (all *p* for interaction >0.05).

**Table 3.** Fully Adjusted Cox Regression Models for Long-Term Mortality Among 90-Day Stroke Survivors, Using Multiply Imputed Data

​	Ischemic stroke		Intracerebral hemorrhage	
Variable^a^	aHR (95% CI)	*p* value	aHR (95% CI)	*p* value
mRS 3^b^ (vs. 0–2)	2.05 (1.86–2.26)	<0.001	1.33 (0.98–1.80)	0.071
mRS 4 (vs. 0–2)	3.57 (3.20–3.98)	<0.001	2.55 (1.89–3.43)	<0.001
mRS 5 (vs. 0–2)	5.69 (5.03–6.43)	<0.001	3.92 (2.86–5.37)	<0.001
Year 2020 (vs. 2013)^c^	0.86 (0.80–0.92)	<0.001	0.98 (0.80–1.19)	0.801
Age (per year)	1.06 (1.06–1.06)	<0.001	1.05 (1.04–1.06)	<0.001
Female sex	0.74 (0.68–0.79)	<0.001	0.93 (0.76–1.14)	0.508
Diabetes mellitus	1.21 (1.12–1.32)	<0.001	1.16 (0.90–1.50)	0.247
Hypertension	1.02 (0.94–1.10)	0.719	1.02 (0.82–1.27)	0.865
Atrial fibrillation	1.38 (1.27–1.50)	<0.001	0.98 (0.71–1.35)	0.885

^a^ Models were adjusted for age, sex, diabetes mellitus, hypertension, atrial fibrillation, calendar year (2020 vs. 2013), and 90-day functional outcome.

^b^ mRS refers to functional status at 90 days after stroke.

^c^ Calendar-year cohort is the exposure of interest. Covariates are shown to document adjustment and should not be interpreted as independent causal effects.

**Fig. 4.**
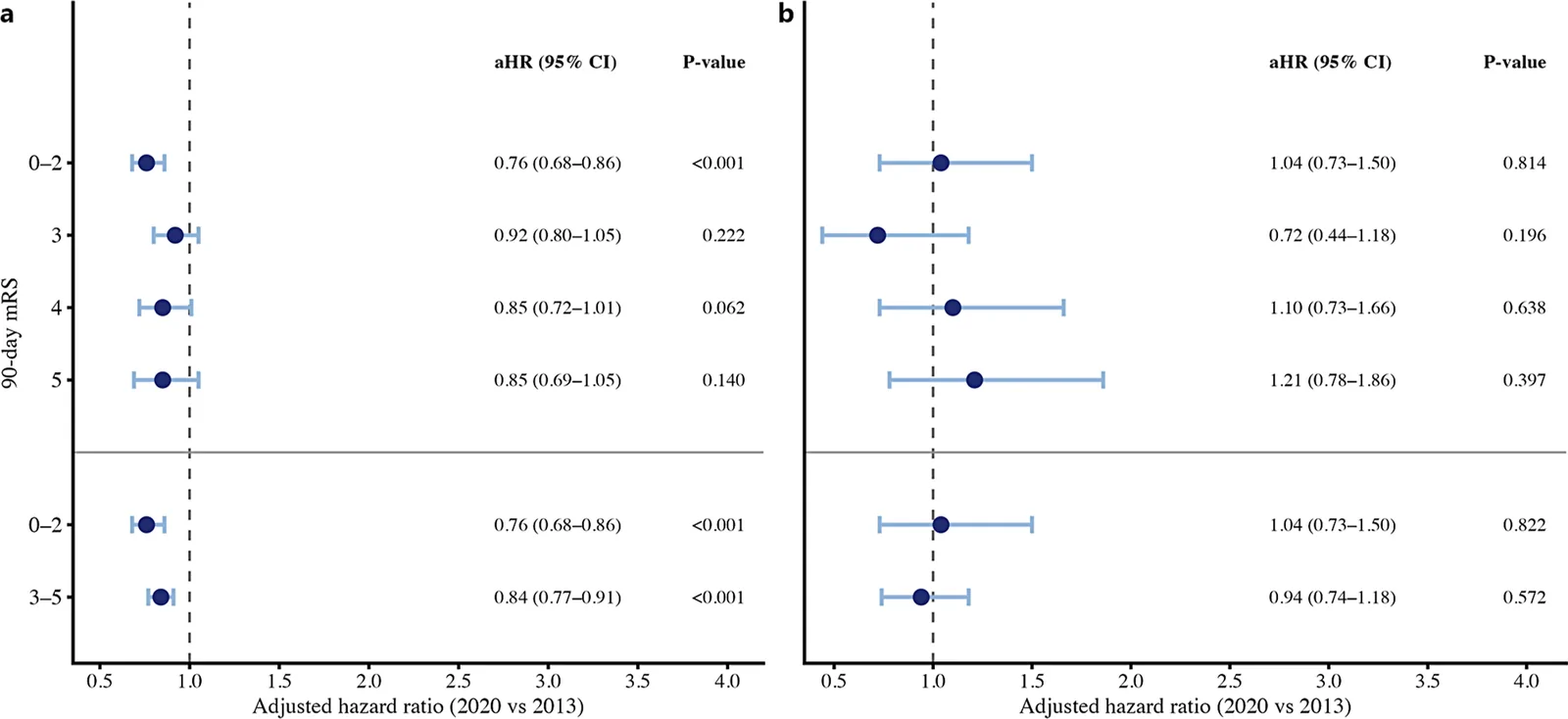
Adjusted association between calendar-year cohort and mortality from day 90 to 3 years within strata of 90-day mRS. Forest plot showing adjusted hazard ratios (aHRs) for long-term mortality comparing 2020 versus 2013 within each 90-day modified Rankin Scale (mRS) category. Panel (**a)** shows ischemic stroke and panel** (b)** intracerebral hemorrhage. Estimates were derived from multivariable Cox regression models with an interaction term between calendar year and 90-day mRS and were pooled across multiply imputed datasets using Rubin’s rules. Dichotomized Cox regressions are shown below the horizontal line.

Sensitivity analyses restricted to complete cases yielded effect estimates comparable to those obtained from the multiply imputed models in both the main Cox regression and the stratified analyses (online suppl. Tables S4 and S5). Complete-case Cox regression analyses included 20,704 patients with IS and 2,211 patients with ICH. In analyses additionally adjusted for baseline stroke severity using NIHSS (online suppl. Table S7), the association between calendar year and long-term mortality remained largely unchanged. Among patients with IS, calendar year 2020 was associated with lower mortality both among patients with mRS 0–2 at 90 days (aHR 0.72, 95% CI 0.61–0.84) and among those with mRS 3–5 (aHR 0.80, 95% CI 0.70–0.90). No statistically significant interaction between calendar year and 90-day disability strata was observed. Sequential addition of previous stroke and current smoking produced similar calendar-year estimates to the original complete-case model in both stroke subtypes (online suppl. Table S8). Among patients with IS, complete-case aHRs for 2020 versus 2013 were 0.81 (95% CI 0.74–0.90) without AF and 0.84 (0.74–0.95) with AF (*p* for interaction = 0.565). Associations remained in the same direction after NIHSS adjustment (online suppl. Table S9). Among patients with IS and AF, median admission NIHSS was 4 (IQR 1–9) in 2013 and 4 (1–8) in 2020 without OAC, and 2 (1–6) in both years with OAC (online suppl. Table S10).

## Discussion

In this nationwide comparison of two Riksstroke cohorts of prestroke-independent 90-day stroke survivors, mortality from day 90 to 3 years after IS was lower in the 2020 cohort than in the 2013 cohort. Notably, improved survival was observed both in those with favorable functional outcome and in those with residual disability at 90 days, indicating that gains in long-term survival extend across all levels of poststroke disability. In contrast, no significant improvement in long-term survival was observed across 90-day disability strata after ICH between the observed calendar years.

While previous studies have demonstrated increased survival after IS [2–4], it remains unclear whether such improvements are distributed across levels of poststroke disability. Our findings address this gap by showing that survival gains are not confined to those with favorable recovery. In addition, although 90-day mRS is a well-established predictor of long-term prognosis [5–9], our findings show that its prognostic value is preserved in contemporary stroke care, with consistent associations observed across two cohorts and improvements in survival evident across all functional outcome strata in 2020. These findings support the continued use of 90-day mRS as a clinically meaningful endpoint in stroke studies, given its strong association with long-term survival.

By restricting the analysis to patients alive at 90 days and stratifying by functional outcome at that time point, the present study addresses changes in survival in the post-acute phase. This approach reduces the influence of early mortality and acute stroke severity and allows a more direct assessment of factors influencing long-term prognosis. The observation that survival improved across disability levels suggests that these gains are unlikely to be explained solely by acute stroke treatments and instead may reflect changes in post-acute management. This interpretation is supported by the comparison of baseline characteristics within mRS strata (online suppl. Table S6), which showed no major differences in age, stroke severity, or overall vascular risk burden between 2013 and 2020. The absence of substantial shifts in case mix within functional outcome groups argues against systematic selection of lower-risk patients into the later cohort. Instead, the changes observed may plausibly be related to treatment patterns, including increased use of OACs and statins for secondary prevention and greater access to stroke unit care. A reduction in recurrent stroke could also have contributed to lower mortality after IS. However, longitudinal data on recurrent stroke across the full post-90-day follow-up period were not available, preventing direct evaluation of this explanation.

We observed substantial increases in OAC use among patients with AF, particularly in those aged ≥80 years, where treatment rates increased markedly (Fig. 1). This shift was largely driven by increased use of direct OACs, which have been shown to be effective and safe in older populations and are now more widely implemented in clinical practice during the last decade [23]. The exploratory AF/OAC analyses did not identify a change in median NIHSS within OAC strata, but substantial, differential NIHSS missingness and selection of prestroke-independent 90-day survivors limit inference about changes in severity. These analyses cannot establish a causal effect of preventive treatment on severity or subsequent survival. Similarly, statin use increased particularly among older patients (Fig. 1). These changes are consistent with improved implementation of guideline-recommended secondary prevention, especially in older patients, but the present study cannot determine whether specific treatment changes mediated the observed between-cohort difference in survival [24]. This interpretation is consistent with previous findings suggesting that survival gains between 2013 and 2020 have been most pronounced among older individuals, particularly those aged ≥75 years [10]. Although mortality increased progressively with greater 90-day disability in both IS and ICH, apparent differences between stroke subtypes or disability strata should be interpreted cautiously. Complications related to immobility, rehabilitation, and the intensity of healthcare or social support may influence long-term prognosis, but these mechanisms could not be evaluated in the present dataset.

In contrast, no improvement in long-term survival was observed after ICH. This divergence between stroke subtypes is consistent with previous studies reporting limited progress in outcomes after ICH [3, 25] and supports the interpretation that advances in secondary prevention and long-term management, rather than general improvements in healthcare delivery, are key drivers of the observed survival gains after IS.

### Strengths

This study has several strengths. First, restriction to prestroke-independent patients (mRS 0–2) improved interpretability of poststroke functional strata in a post-acute survivor cohort. Second, parallel analyses of IS and ICH provided a comparison of outcomes in two major stroke subtypes within the same healthcare system and calendar periods. Third, the large nationwide cohort with high registry coverage enhanced statistical precision. Finally, although missingness in 90-day mRS was substantial, multiple imputation using strong predictors of functional outcome, including discharge destination, appeared feasible, and findings were consistent with complete-case analyses.

### Limitations

Several limitations should be acknowledged. First, restriction to prestroke-independent patients improves interpretability but limits generalizability to patients with prestroke dependency, disability, or frailty. This is important because comorbidity, disability, and frailty are distinct but interrelated constructs in stroke and may influence care pathways, prognosis, and response to interventions [26]. Riksstroke is also hospital based and does not capture strokes that do not lead to hospital admission. Characteristics and outcomes of nonregistered patients were unavailable, so differential selection between cohorts cannot be excluded. Second, estimates in the ICH cohort, particularly within higher mRS strata, were less precise, as reflected by wider CIs, and stratified analyses within higher disability levels after IS were also estimated with lower precision due to fewer observations. The present analysis also combined mRS categories 0–2 into a single group reflecting functional independence, precluding assessment of potential differences within the lower disability range. Third, stroke severity, as assessed by NIHSS, had a substantial proportion of missing data; although sensitivity analyses incorporating NIHSS yielded estimates consistent with the main analyses, the degree of missingness limits the ability to fully account for differences in baseline stroke severity between cohorts, and a more favorable case mix in 2020 cannot be entirely excluded as a contributing factor. Fourth, the proportion of missing data was also substantial for 90-day mRS, particularly in ICH and in the 2020 cohort; multiple imputation was applied and sensitivity analyses yielded similar results. Fifth, comparisons across calendar years may also be affected by secular changes in healthcare delivery and background mortality, and the COVID-19 pandemic during 2020 may have influenced both healthcare-seeking behavior and access to care, with potential underrepresentation of milder stroke cases and altered management of more severe cases. Swedish pandemic measures in 2020 included restrictions on public gatherings and extensive public-health advice and recommendations aimed at reducing social contacts and transmission. Measures were further intensified during the later part of 2020 [27]. This is an additional reason to interpret the findings as a comparison of two discrete cohorts rather than as a generalizable continuous temporal trend. Confirmation in a post-pandemic cohort would be valuable to determine whether these findings persist under more typical healthcare conditions. Sixth, data on socioeconomic factors, including education, migration status, and access to rehabilitation, were not available and may represent unmeasured confounding in the present analyses. Temporal changes in diagnostic intensity and the recording of comorbidities could also contribute to residual confounding. Recorded risk factors did not increase uniformly between cohorts, but this does not establish comparable ascertainment. Similar estimates after additional adjustment for previous stroke and smoking are reassuring, although these complete-case analyses do not exclude selection bias or unmeasured confounding. Finally, as in all observational registry-based studies, residual confounding cannot be excluded despite adjustment for several clinically relevant variables. Importantly, the study does not by itself establish causality, and any causal interpretation depends on assumptions that cannot be fully verified in this observational registry-based comparison. The present analyses cannot determine which specific factors contributed to the observed between-cohort differences in survival.

### Future Directions

Future studies should aim to further elucidate the mechanisms underlying the observed improvements in long-term survival after IS. In particular, studies incorporating detailed longitudinal data on secondary prevention, recurrent vascular events, and adherence to medical therapy are needed to better understand the relative contribution of different post-acute interventions. In addition, analyses including more granular functional outcome measures, with separation of mRS 0, 1, and 2, may help clarify whether survival gains differ within the spectrum of mild disability. Given the absence of improvement in ICH, there is also a clear need for studies focusing on long-term outcomes and secondary prevention strategies in this patient group. Finally, future research should explore whether similar patterns are observed in other healthcare settings to assess the generalizability of these findings.

## Conclusion

In conclusion, post-90-day survival after IS was higher in the 2020 cohort than in the 2013 cohort across the spectrum of poststroke disability, including among patients with residual disability at 90 days. These findings suggest that improvements in post-acute care and secondary prevention may have contributed to improved long-term survival. No corresponding improvement was observed after ICH, highlighting the need for more effective long-term management strategies in this population. Confirmation in other populations, including post-pandemic cohorts, and investigation of the underlying mechanisms are warranted.

## Acknowledgments

The authors are grateful to Riksstroke for providing access to the data used in this study, as well as the patients and healthcare professionals who contributed to the register.

## Statement of Ethics

The study was approved by the Swedish Ethical Review Authority (Dnr 2024-08008-01) and was conducted in accordance with the Declaration of Helsinki. The requirement for individual informed consent was waived because the study was based on retrospective analysis of pseudonymized registry data, in accordance with Swedish legislation and the ethical approval for the study.

## Conflict of Interest Statement

B.N. having received honoraria from Moleac and Merck and serves as an Associate Editor and Review Board Member for *Neuroepidemiology*. T.U. has received speaker honoraria from Siemens Healthineers. T.A.-H. has received speaker honoraria from Chiesi Pharmaceuticals. The remaining authors declare no potential conflicts of interest.

## Funding Sources

This work was supported by Regional ALF grants (T.U., B.M.H., and T.A.-H.), SUS Stiftelser & Fonder (B.M.H.), the Swedish Heart-Lung Foundation (T.A.-H.), and the Bundy Academy (T.U. and T.A.-H.). The funding sources had no role in the study design, data collection, data analysis, or interpretation of the results.

## Author Contributions

All authors contributed to the conception and design of the study. Data management and statistical analyses were performed by M.J. and M.P. All authors contributed to the interpretation of the findings. The study was supervised by T.A.-H., T.U., M.P., B.N., and B.M.H. M.J. drafted the initial manuscript. All authors critically reviewed and revised the manuscript for important intellectual content and approved the final version for submission.

## Data Availability

The individual-level data underlying this study are not publicly available because they contain sensitive registry data. Access to Riksstroke data may be granted following application to the register holder and is subject to relevant ethical, legal, and data protection requirements.
